# Inhibitory Effect of Curcumin-Cu(II) and Curcumin-Zn(II) Complexes on Amyloid-Beta Peptide Fibrillation

**DOI:** 10.1155/2014/325873

**Published:** 2014-07-23

**Authors:** Rona Banerjee

**Affiliations:** Department of Biotechnology, Indian Institute of Technology Roorkee, Roorkee, Uttarakhand 247667, India

## Abstract

Mononuclear complexes of Curcumin with Cu(II) and Zn(II) have been synthesized and, characterized and their effects on the fibrillization and aggregation of amyloid-beta (A*β*) peptide have been studied. FTIR spectroscopy and atomic force microscopy (AFM) observations demonstrate that the complexes can inhibit the transition from less structured oligomers to *β*-sheet rich protofibrils which act as seeding factors for further fibrillization. The metal complexes also impart more improved inhibitory effects than Curcumin on peptide fibrillization.

## 1. Introduction

Over the years, Curcumin [1, 7-bis(4-hydroxy-3methoxyphenyl)-1, 6-heptadiene-3, 5-dione] has emerged as a multifunctional phytochemical with antioxidant, anti-inflammatory, anticancer, and neuroprotective properties [1–5]. However, in neurodegenerative diseases like Alzheimer's disease (AD) the molecular mechanism of action of Curcumin is not well understood. Amyloid beta (A*β*) is a peptide of 36–42 amino acids that is processed from a transmembrane glycoprotein called amyloid precursor protein (APP), and the A*β*42 is known as the most neurotoxic variant in the pathology of AD. The misfolding of A*β* is governed by a number of microenvironmental parameters and is primarily responsible for aggregation of the peptide which may lead to neuronal cell death and cognitive impairment. The aggregation process is initiated by oligomerization of the soluble monomers, followed by their association into protofibrils and finally the bunch of fibrils forms the amyloid plaques [6]. Biometals like Cu(II) and Zn(II) are found in abundance in the synaptic area of AD brain and dysregulation of metal homeostasis can lead to binding of the free metal ions with A*β* forming metal-peptide complexes that accelerate the peptide aggregation [7]. Such metal-peptide complexes can, therefore, become seeding factors in the amyloid plaque formation. Oxidative stress and generation of reactive oxygen species (ROS) also play crucial roles in accelerating the peptide fibrillization which in turn can generate more ROS causing a deleterious vicious cycle of neurodegeneration [8, 9]. Earlier reports suggest that copper ions entrapped in A*β* fibrils are electrochemically active and can generate ROS depending on the microenvironment [10, 11]. Therefore, metal chelation by small antioxidant compounds like Curcumin and other phytochemicals could effectively contribute to the development of potential therapeutic strategies for protein misfolding diseases. Curcumin has two o-methoxy phenolic OH groups attached to the *β*-diketone (heptadiene-dione) moiety that can form chelates of the type 1 : 1 (ML) and 1 : 2 (ML2) with copper, iron, and other transition metals. Although the metal chelation may occur through both the o-methoxy phenol and the *β*-diketo group, in most of the cases, the complexation of Curcumin with metal ions involves the diketo group [12, 13]. Several metallocomplexes of Curcumin have been synthesized, characterized, and evaluated for various biological activities which imply the importance of the free phenolic OH groups of Curcumin for scavenging free radicals and reducing cytotoxicity [14–16]. Yang and coworkers have shown that Curcumin by itself can inhibit the oligomerization and aggregation of the A*β* peptide* in vivo* and increase neuronal cell viability in a dose-dependent manner [17]. Our present study addresses the possibility of beneficial action of Curcumin-metal complexes on the aggregation and fibrillization of amyloid peptide. I have synthesized and characterized the mononuclear Curcumin-Cu(II) and Curcumin-Zn(II) complexes. The formation of fibrils and amyloid aggregates by the A*β*42 peptide in phosphate buffer saline (PBS) at physiological pH in presence of Curcumin and the metal complexes have been studied by atomic force microscopy (AFM). The effect of Curcumin and the complexes on the alterations of secondary structure of the peptide was investigated by FTIR spectroscopy which also indicates antiamyloidogenic property of the Curcumin-metal complexes.

## 2. Materials and Methods

Curcumin (99% pure), spectroscopic grade acetone, ethanol, and dimethyl sulfoxide (DMSO) were procured from Merck (Germany); copper acetate, zinc acetate, and other salts for buffer preparation were purchased from Loba Chemie and Himedia. The 10 mM phosphate buffered saline (PBS) was prepared in Millipore water and pH of the buffer was adjusted to 7.4 in the working solutions. Concentrated stock solution of Curcumin was prepared in acetone. The lyophilized A*β*42 peptide (human) of 95% purity (Genscript, USA) was used without any further modification. The synthetic peptide was dissolved in slightly alkaline PBS (pH 8) by adding 0.1 M NaOH solution as required to obtain monomeric soluble peptide without preseeded fibrils and the stock was stored at −20°C. For AFM studies, the peptide was dissolved in a low salt phosphate buffer.

Mononuclear Curcumin-Cu(II) complex was synthesized following the protocols by Barik et al. [15]. For the synthesis of mononuclear Curcumin-Zn(II) complex we have adopted the method reported by Zhao et al. [18]. The reddish orange colored Curcumin-Zn(II) complex and dark brown Curcumin-Cu(II) complexes were obtained which are soluble in DMSO and are also stable in DMSO-water mixture. The stability of Curcumin and the metal complexes was checked in working solutions before every experiment. Electrospray ionization mass spectra (ESI-MS) of Curcumin-metal complexes were recorded on microTOF-Q2-10328 instrument to determine their molecular mass. The photophysical properties of the complexes were characterized by UV absorption and FTIR spectroscopy.

Varian-Cary100 spectrophotometer was used for acquiring the absorption spectra using a pair of black walled suprasil quartz cuvettes (from Hellma, Germany) with 10 mm path length. Solid state FTIR spectra in transmission mode were recorded on a Nicolet NEXUS Agilent 1100 FT-IR Spectrometer, in the 400–4000 cm^−1^ wavenumbers range using KBr pellets. For each spectrum, water vapour subtraction and baseline corrections were done. To obtain the final FTIR spectrum, 100 interferograms were coadded and Fourier-transformed. Atomic force microscope (AFM, NT-MDT model NTEGRA T5-150) was employed for imaging of the peptide in the semicontact mode at ambient air (RH 50%) and temperature (25°C) with a silicon cantilever at its resonance frequency. The samples for microscopic imaging were prepared on clean glass coverslips in very thin layers to avoid any distortion by the cantilever tip. The samples on the coverslips were dried at room temperature for 3 hours in a closed petri dish before mounting to the microscope. At least five successive scans were taken for each image. When stable images were obtained, the scanning force was minimized by a reduction of the set point voltage, and the scanning area was increased to the desired size.

## 3. Results and Discussion

**Figure 1:**
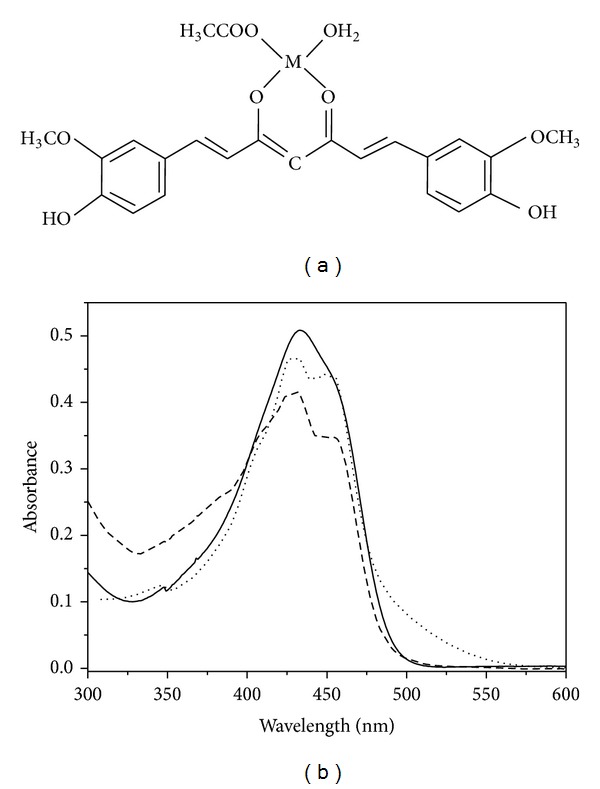
(a) Chemical structures of mononuclear Curcumin-metal complex showing the *β*-diketone moiety of Curcumin as the metal binding site and (b) UV-vis absorption spectra of Curcumin (—), Curcumin-Cu(II) complex (- - -), and Curcumin-Zn(II) complex (*····*).

**Figure 2:**
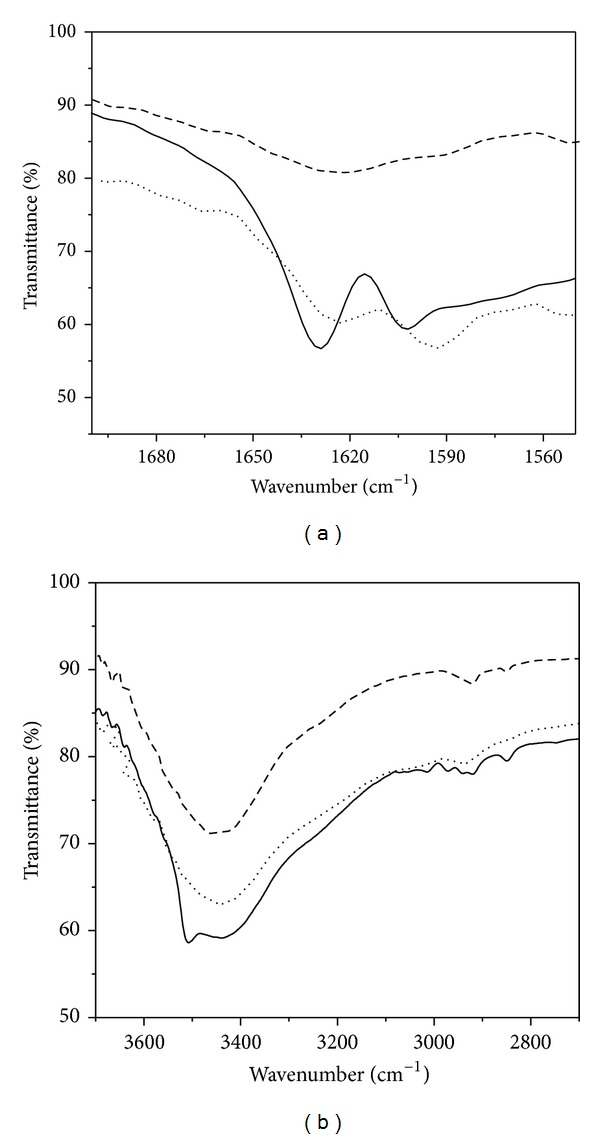
FTIR spectra of (a) 1500–1700 cm^−1^ region and (b) 2800–3800 cm^−1^ region of Curcumin (—), Curcumin-Cu(II) complex (- - -), and Curcumin-Zn(II) complex (*····*).

The general chemical structure of the mononuclear (ML type) Cu(II) and Zn(II) complexes of Curcumin has been shown in Figure 1(a) considering the *β*-diketone moiety of the ligand as the conjugation site for the metal ions. The molecular ion peaks for Curcumin-Cu(II) complex and Curcumin-Zn(II) complexes were positioned at 508.06 and 509.06 mass units, respectively, in the ESI-MS spectra (see Supplementary Data in Supplementary Material available online at http://dx.doi.org/10.1155/2014/325873) indicating that the acetate ions are one of the additional ligands rather than the hydroxyl group in the ML type complexes. The molecular formula for the metal complexes was deduced as C_21_H_19_O_6_M·OCOCH_3_·H_2_O. The difference of m/z values from that expected from the brutto formula for the copper and zinc complexes was 0.08 and 0.76, respectively. The binding stoichiometry of Curcumin: metal in solution has also been confirmed to be 1 : 1 by mole ratio method using absorption spectroscopy.  Figure 1(b) shows the absorption spectra of Curcumin and its metal complexes in DMSO.Curcumin exhibits the absorption maximum (*λ*max) at 433 nm, due to *π* − π* transition, with a shoulder at 453 nm. For the Curcumin-metal complexes, the *λ*max of Curcumin undergoes a 5 nm blue shift with distinct appearance of vibronic bands around 410 nm and 450 nm that can be attributed to the Curcumin-metal charge transfer indicating the involvement of the carbonyl group of Curcumin in complexation with the metals. The ground state spectral features of the Curcumin-Cu (II) complex are consistent with that of the 1 : 1 ML complex reported by Barik et al. [15] and Zebib et al. [19] but are distinctly different from that of the 2 : 1 Curcumin-Cu(II) complex reported in the literature [20]. The extinction coefficients at 425 nm for the Cu(II) and Zn(II) complexes in DMSO at various concentrations of the complexes were determined from the plots of concentration versus absorbance at 425 nm (Supplementary Data). The values of extinction coefficients obtained for Curcumin-Cu(II) and Curcumin-Zn(II) complexes were 92730 M^−1^cm^−1^ and 83930 M^−1^ cm^−1^, respectively. Further characterizations of the metal complexes were done by FTIR spectroscopy. The FTIR spectra of Curcumin and that of the metal complexes show that the *ν*C=O stretching band of Curcumin at 1628 cm^−1^ is shifted to a lower wavenumber, that is, 1621 cm^−1^ on complexation with Cu(II) and Zn(II) (Figure 2(a)), implying that the conjugation of the metal ions takes place through the *β*-diketone moiety of Curcumin. However, the position of vibrational band around 3450 cm^−1^ of Curcumin remains unchanged on complexation with metal ions (Figure 2(b)). Literature data suggest that the phenolic OH group of Curcumin is not involved in complexation with other metal ions exhibiting unaltered vibrational band around 3500 cm^−1^ in the FTIR spectra of Curcumin-metal complexes [14, 21]. Again, the enolic OH of Curcumin is primarily important for its antioxidant and free radical scavenging property [14–16, 22]. Consistent with the previous reports, my observations lead to the conclusion that the binding of Cu(II) and Zn(II) does not involve the phenolic OH group of Curcumin causing no loss of antioxidant property of the ligand molecule.

**Figure 3:**
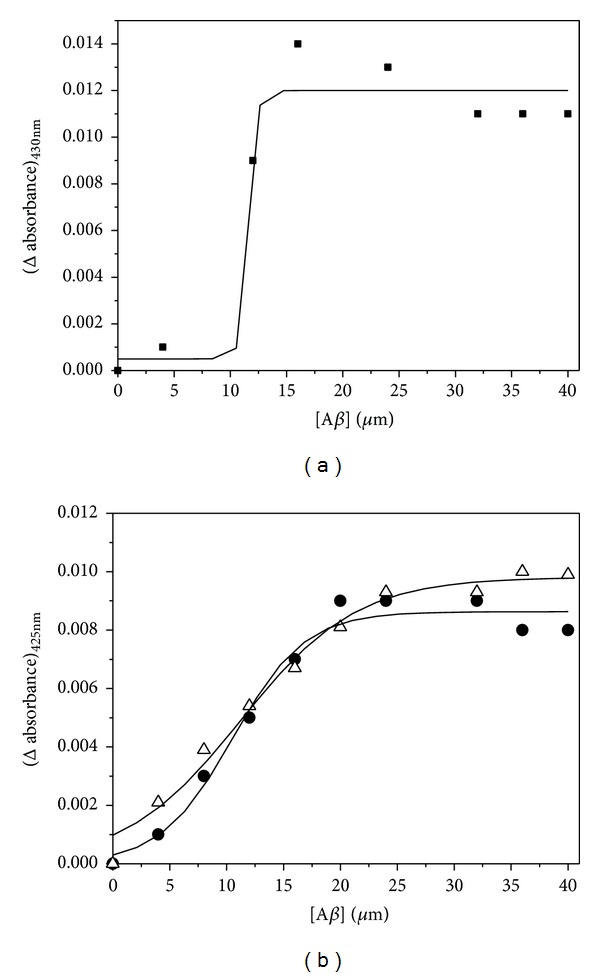
(a) Plot of difference in absorbance at 430 nm for Curcumin monitored as a function of A*β* peptide concentration. (b) Plot of difference in absorbance at 425 nm for Curcumin-Cu(II) complex (filled circles) and Curcumin-Zn(II) complex (open triangles) with increasing peptide concentration in buffer.

In order to understand the interaction of the complexes with A*β* peptide, the binding isotherms of monomeric, soluble peptide have been studied by observing the changes in absorption at 430 nm for Curcumin and at 425 nm for Cu(II) complex and Zn(II) complex (Figure 3) on gradual addition of peptide to 20 *μ*M of Curcumin and the complexes separately. The increase in absorbance upon increasing concentration of the peptide reflects the association of the soluble monomeric form of the peptide with Curcumin and the metal complexes. The half saturation values of peptide for complexation with Curcumin, Curcumin-Cu(II), and Curcumin-Zn(II) were determined as 11.6 *μ*M, 10.2 *μ*M, and 12.3 *μ*M, respectively. These values indicate that Curcumin and both the complexes have similar binding affinity with the soluble monomeric form of A*β* peptide.

**Figure 4:**
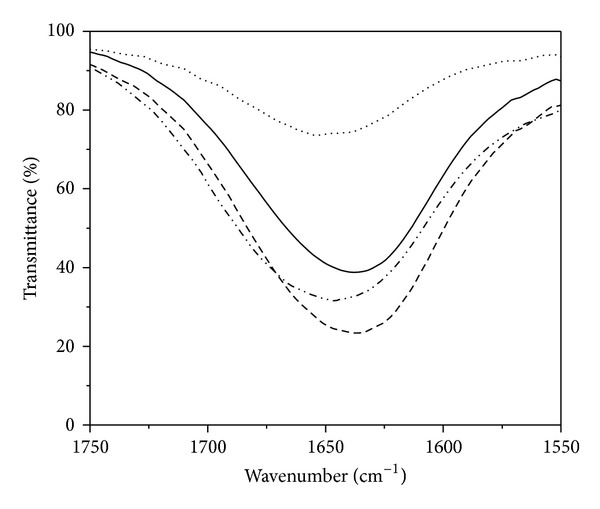
FTIR spectra of the amide-I region for A*β* peptide alone (—) and that with Curcumin (- - -), with Curcumin-Cu(II) complex (*····*), and with Curcumin-Zn complex (-*··*-*··*-*··*). The concentration of the peptide was 30 *μ*M and that of Curcumin, Curcumin-Cu(II), and Curcumin-Zn(II) complexes was 100**** *μ*M in the respective samples.

The effect of Curcumin and the complexes on the secondary structure of the peptide has been investigated by FTIR spectroscopy. In case of peptides, the IR absorption is sensitive to the backbone secondary structure and gives useful information regarding the conformation of the peptide irrespective of the amino acid sequence. The amide-I and the amide-II bands are the two major regions of protein infrared spectrum. The amide-I band (between 1600 and 1700 cm^−1^) is mainly associated with the C=O stretching vibration and could be directly correlated with the backbone conformation. Amide-II results from the N-H bending vibration and from the C-N stretching vibration. 30 *μ*M A*β* solution in PBS (pH 7.4) in four aliquots was prepared of which the first one is the untreated peptide, that is, peptide in buffer, and the other three are that with added Curcumin and the Cu(II) and Zn(II) complexes separately (100 *μ*M each). The FTIR spectra of the above samples were shown in Figure 4. The backbone sensitive amide-I vibrational band appears at 1633 cm^−1^ for the untreated A*β* peptide and at 1638 cm^−1^ for Curcumin treated sample. As the band frequency around 1630 cm^−1^ is characteristic of *β*-sheet structure prevailing in fibrils and a shift to lower wavenumbers corresponds to higher content of *β*-sheets, therefore, it is evident from the FTIR spectra that incorporation of Curcumin in the buffer could slightly reduce the *β*-sheet content. For the peptide samples treated with Curcumin-metal complexes the amide-I band undergoes an appreciable shift to higher wavenumbers. The amide-I bands are positioned at 1650 cm^−1^ and 1645 cm^−1^, respectively, for A*β* peptide in association with the Curcumin-Cu(II) and Curcumin-Zn(II) complex. Earlier report by Ahmed et al. [6] suggests that, for the less structured oligomers, the amide-I band appears around 1645 cm^−1^ whereas the band at 1630 cm^−1^ is the signature of more ordered *β*-sheet rich fibrillar structure. Similarly, Mastrangelo et al. [23] have shown that the strong band around 1630 cm^−1^ corresponds to *β*-sheet secondary structure formed rapidly in the aqueous solvent. My observations from FTIR study, therefore, imply that the native peptide had a high content of *β*-sheets structure that could act as seeding factor for further fibrillization. On incorporation of Curcumin in peptide sample, the *β*-sheet content was slightly reduced, but both the metal complexes, specially the Curcumin-Cu(II) complex, could remarkably inhibit the transition from oligomeric to protofibril structure of the peptide.

**Figure 5:**
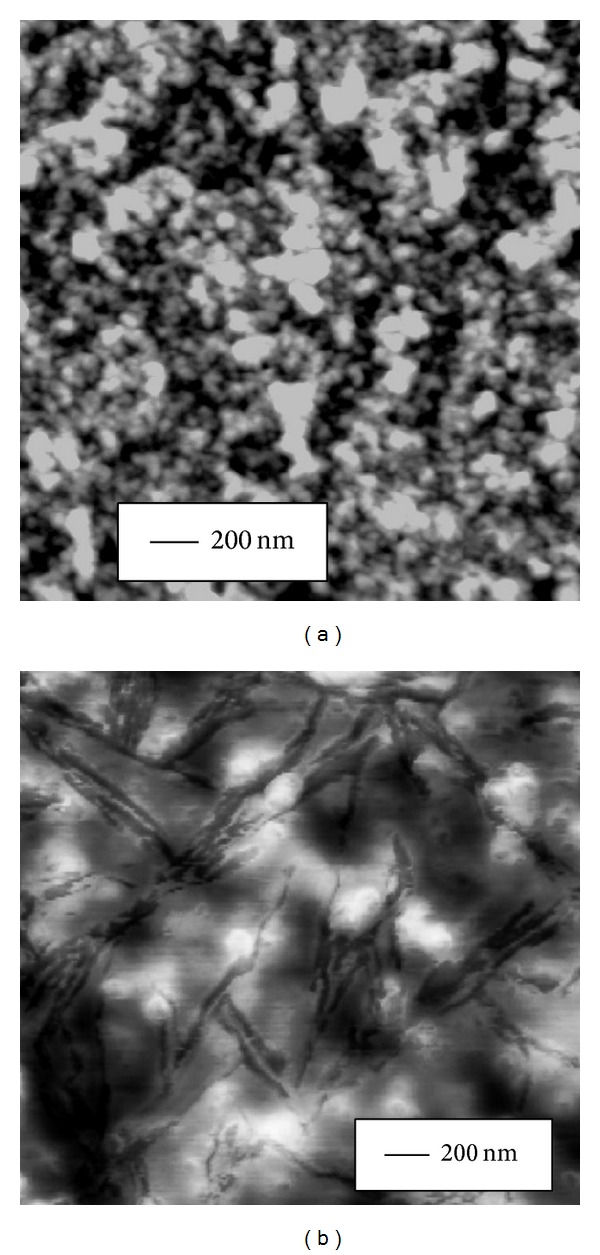
AFM images of 10 *μ*M A*β* peptide at room temperature and in low salt phosphate buffer after incubation for (a) 3 hrs showing oligomeric aggregates and (b) after incubation for 16 days showing the appearance of nanofibrillar structures.

**Figure 6:**
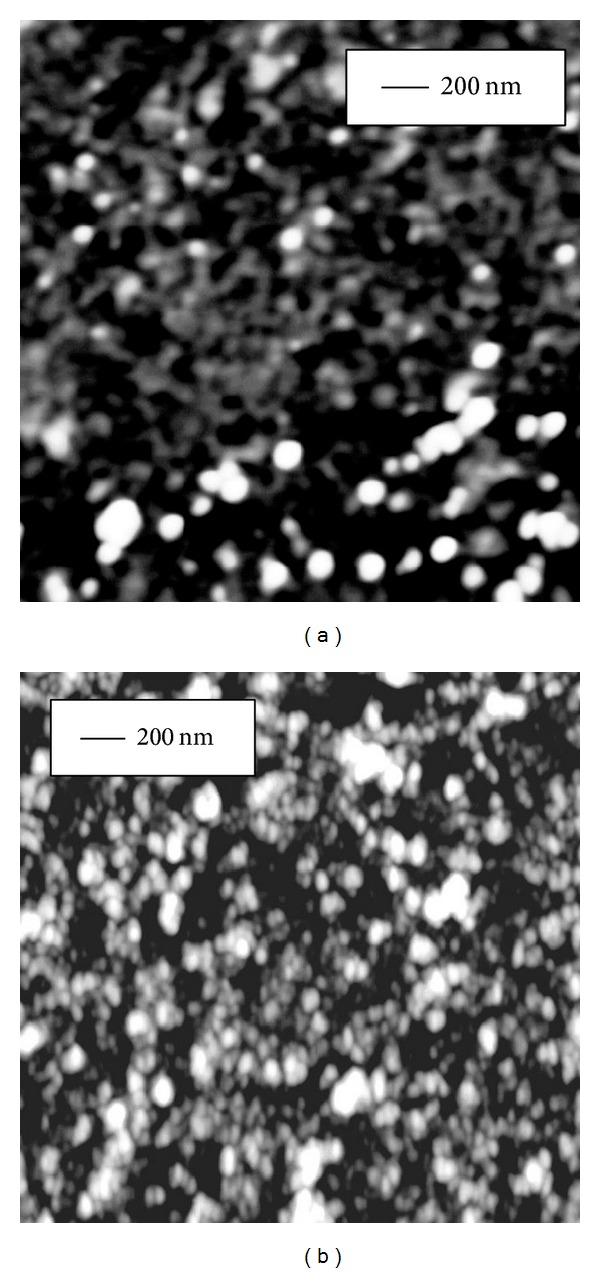
AFM images of amorphous oligomeric assemblies of 10 *μ*M A*β* peptide upon addition of 30 *μ*M Curcumin and after incubation for (a) 3 hrs and (b) 16 days at room temperature. No fibrillar structure was observed on 16 days of incubation.

**Figure 7:**
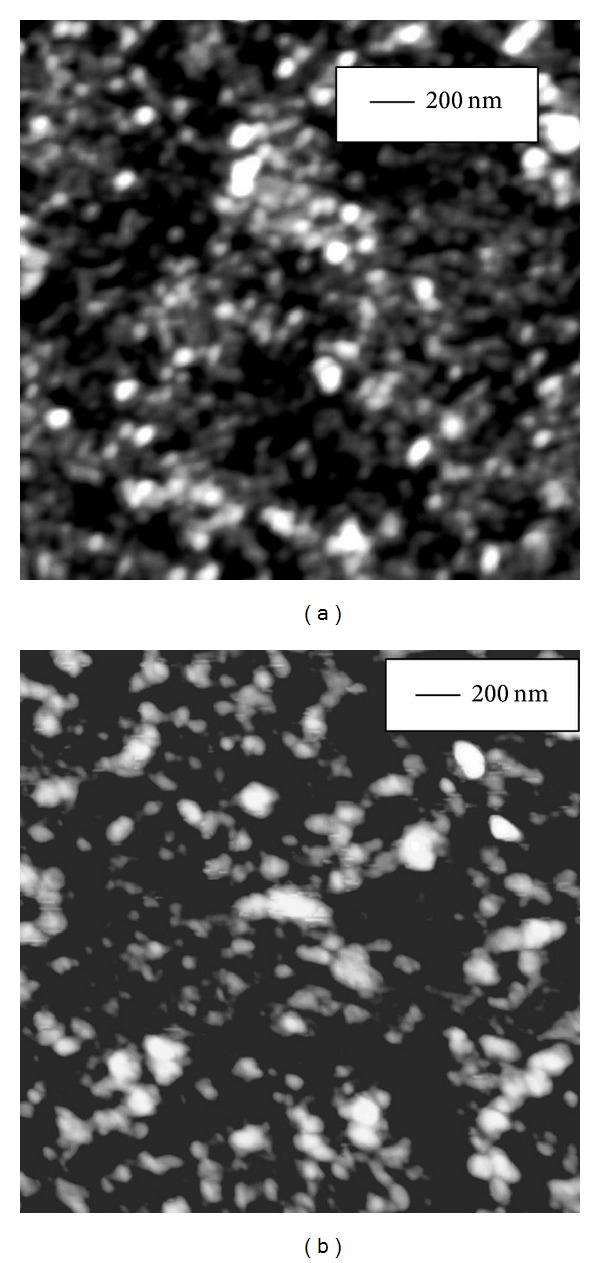
AFM images showing oligomeric assemblies of 10 *μ*M A*β* treated with 30 *μ*M Curcumin-Cu(II) complex after incubation for (a) 3 hrs and (b) 16 days at room temperature. No fibril formation was observed after 16 days of incubation.

**Figure 8:**
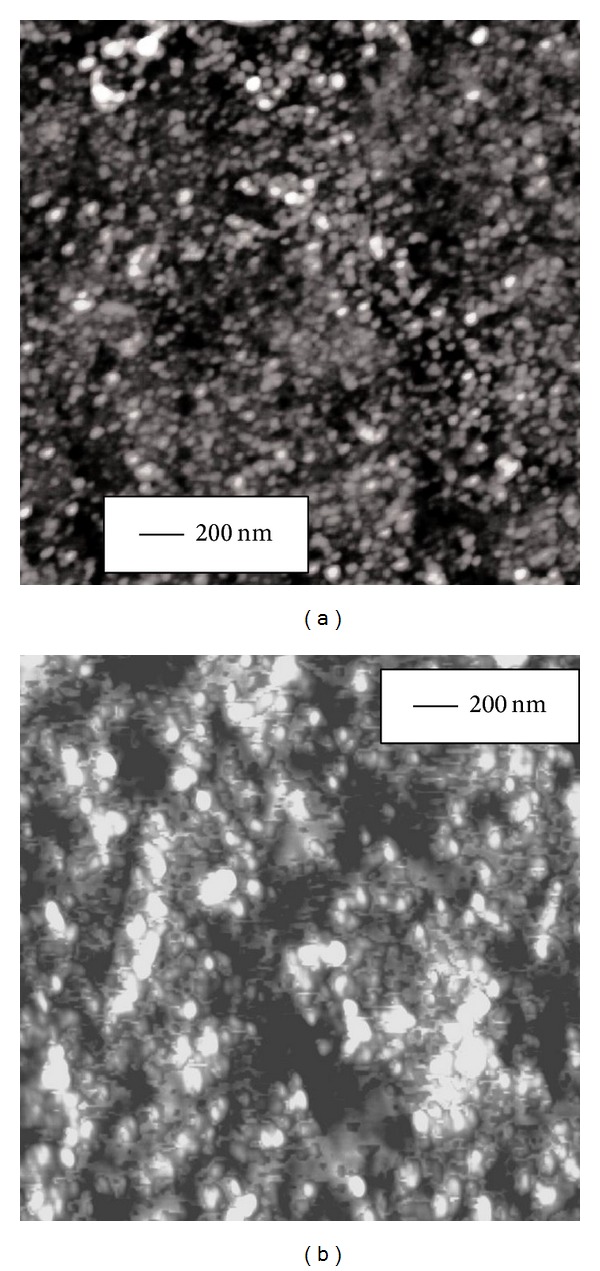
AFM images showing oligomeric assemblies of 10 *μ*M A*β* peptide with 30 *μ*M Curcumin-Zn(II) complex upon incubation for (a) 3 hrs and (b) 16 days at room temperature. Time-dependent fibrillization of peptide was not observed after 16 days of incubation.

The effect of Curcumin and the metal complexes on the secondary structure of peptide backbone was further corroborated by imaging the morphological changes in peptide oligomer assemblies using atomic force microscopy (AFM). Curcumin and the metal complexes were added separately to peptide sample in 3 : 1 mole ratio in low salt phosphate buffer (pH = 7.4) and incubated at room temperature for 3 hours. Amorphous aggregates were detected of average diameter 22*** ***nm for A*β* alone, 16*** ***nm for A*β* with Curcumin, 8*** ***nm for A*β* with Curcumin-Cu(II) complex, and 12*** ***nm for A*β* with Curcumin-Zn(II) complex (Figures 5(a), 6(a), 7(a), and 8(a), resp.). The fibrillization of the peptide was also studied as a function of time using the same samples. Incubation for 16 days at room temperature resulted in the formation of filamentous nanofibrils along with the large oligomeric assemblies in the untreated peptide, whereas no fibrillization is apparent in the samples containing Curcumin and the Curcumin-metal complexes (Figures 5(b), 6(b), 7(b), and 8(b)). The average particle diameters for the oligomeric assemblies were 35 nm, 21 nm, 12 nm, and 14 nm for untreated A*β* and that with Curcumin, Curcumin-Cu(II) complex, and Curcumin-Zn(II) complex, respectively. These observations indicate that time-dependent fibrillization as well as a concomitant increase in the oligomeric aggregates took place for untreated peptide in buffer, whereas absence of any fibrillar structure and smaller size of aggregates imply that Curcumin and the metal complexes not only have inhibited the fibrillization, but also have retarded the polymerization kinetics. The effect was more pronounced for the peptide treated with Curcumin-Cu(II) complex than bare Curcumin and Curcumin-Zn(II) complex which agrees with the FTIR observations for improved inhibitory effect of Curcumin-Cu(II) complex in the oligomers to protofibril transition by amyloid peptide.

Such antifibrillogenic property of Curcumin and the metal complexes could primarily be explained by their ability to scavenge free radicals in the microenvironment, because free radicals and reactive oxygen species are known to accelerate the ageing and aggregation process of the peptide. In previous reports, it was suggested that the phenolic OH group of Curcumin is principally responsible for its free radical scavenging ability and ML type Cu(II) and Zn(II) complexes of Curcumin could also mimic superoxide dismutase (SOD) activity and act as free radical scavengers [14–16]. My FTIR studies of metal complexes have demonstrated that the phenolic OH group of Curcumin is not involved in the complexation with metals. Hence, the Curcumin-Cu(II) and Curcumin-Zn(II) complexes not only retain the antioxidant property of Curcumin but also may possess improved free radical scavenging property than the parent compound. The metal complexes of Curcumin were also proposed to possess better potential to reduce oxidative stress and free radical generation [24] that could significantly affect the amyloidogenesis process of Alzheimer's disease. The differential antifibrillogenic behaviour of the Cu(II) complex and Zn(II) complex could be due to the fact that Cu(II) is redox active and Curcumin-Cu(II) complex has more enhanced antioxidant property than Curcumin-Zn(II) and other metal complexes. The Curcumin-Cu(II) complex was shown to significantly induce cytotoxicity in cancer cells whereas Curcumin-Zn(II) complex imparts moderate cytotoxicity [25]. Therefore, the differential redox activity could also be responsible for their different antioxidant activity. The stoichiometry of metal: ligand and the geometry of the metal complexes are also important for their antioxidant property. Barik et al. [15, 20] have suggested that the ML type Cu(II)-Curcumin complex by virtue of its flexible orthorhombic geometry is more effective antioxidant and SOD mimicking compound than the ML2 complex that has a rigid square planar geometry. Due to better flexibility, the ML complex can undergo more number of redox cycles that accounts for its improved free radical scavenging property compared to the ML2 complex [20]. DFT studies by Addicoat et al. [26] have also suggested that the 2+ oxidation state of copper remains unchanged on complexation with Curcumin and thereby nullifies the possibility of Curcumin to become prooxidant in the presence of copper. Therefore, binding of Curcumin and the metal complexes with the peptide might reduce the generation of free radicals in the microenvironment thereby retarding the formation of seeding aggregates which are crucial factors in the fibrillization. However, the effect of these metal complexes to ameliorate oxidative stress in cells is to be studied for further understanding of their role in the aggregation pathways of amyloid peptide* in vivo*.

## 4. Conclusion

The present studies provide the first ever direct evidence of the antifibrillogenic property of the mononuclear Cu(II) and Zn(II) complexes of Curcumin in physiological buffer solution. The complexes have similar binding affinity to the monomeric A*β* peptide compared to Curcumin. However, the Cu(II) and Zn(II) complexes impart more improved inhibition in the secondary structural transition of the peptide from oligomers to protofibrils than the parent compound and also have improved efficacy to inhibit the fibrillization and aggregation of the peptide. These results may have implications in understanding the molecular mechanism of action of antioxidant-metal complexes in protein misfolding diseases.

## Supplementary Material

Figure (a): Plot of Concentration vs Absorbance (425nm) for Curcumin-Cu(II) complex.
Figure (b): Plot of Concentration vs Absorbance (425nm) for Curcumin-Zn(II) complex.
Figure (c): ESI-Mass spectra of Curcumin-Cu(II) complex.
Figure (d): ESI-Mass spectra of Curcumin-Zn(II) complex.
