# Supplementary material for: Inhibitory Effect of Curcumin-Cu(II) and Curcumin-Zn(II) Complexes on Amyloid-Beta Peptide Fibrillation
Source: Bioinorg Chem Appl. 2014 Jul 23;2014:325873. doi: 10.1155/2014/325873 (PMC4134801; doi:10.1155/2014/325873)
Supplement: Supplementary file 1 — Figure (a): Plot of Concentration vs Absorbance (425nm) for Curcumin-Cu(II) complex. Figure (b): Plot of Concentration vs Absorbance (425nm) for Curcumin-Zn(II) complex. Figure (c): ESI-Mass spectra of Curcumin-Cu(II) complex. Figure (d): ESI-Mass spectra of Curcumin-Zn(II) complex. [file 325873.f1.pdf]

Supplementary files:

(a)

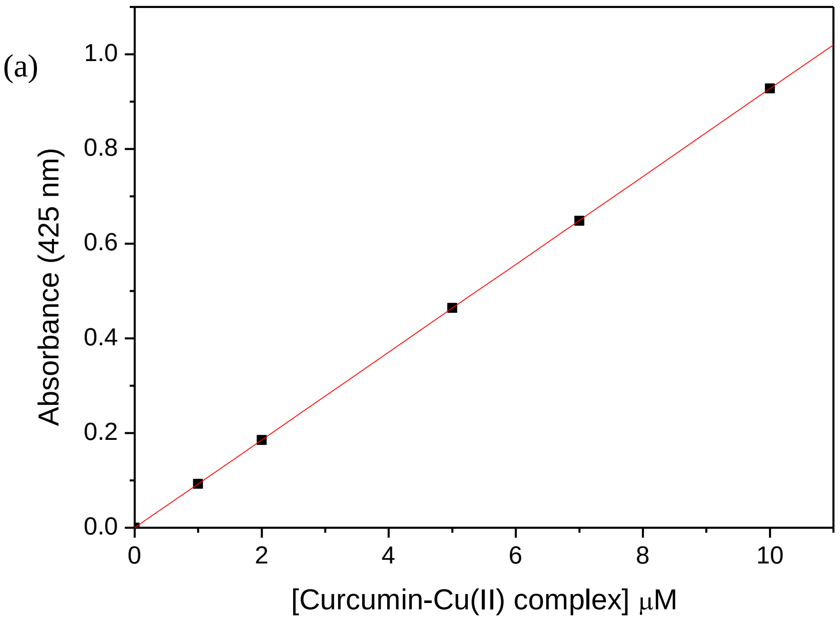

(a) Plot of Concentration vs Absorbance (425nm) for Curcumin-Cu(II) complex.

(b)

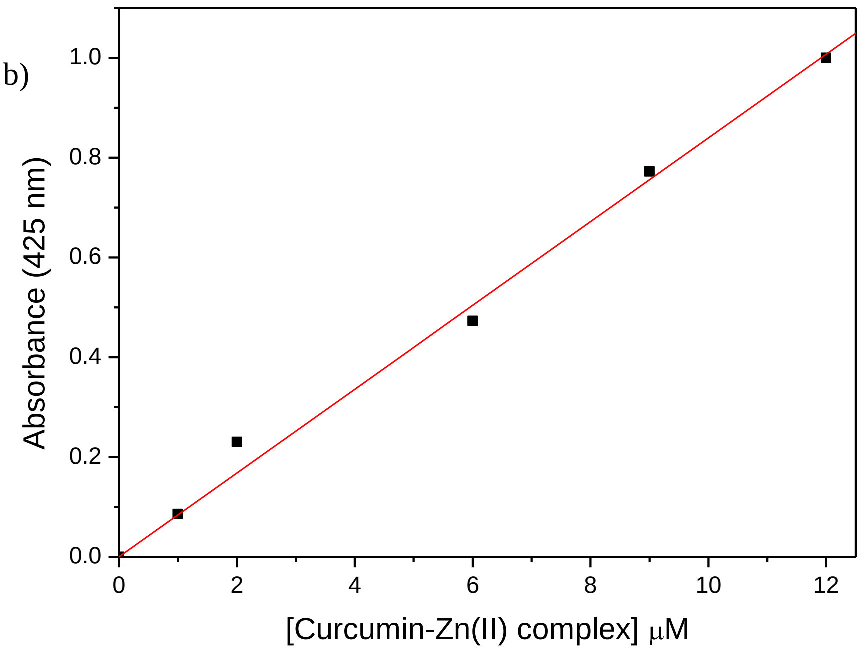

(b) Plot of Concentration vs Absorbance (425nm) for Curcumin-Zn(II) complex.

(c)

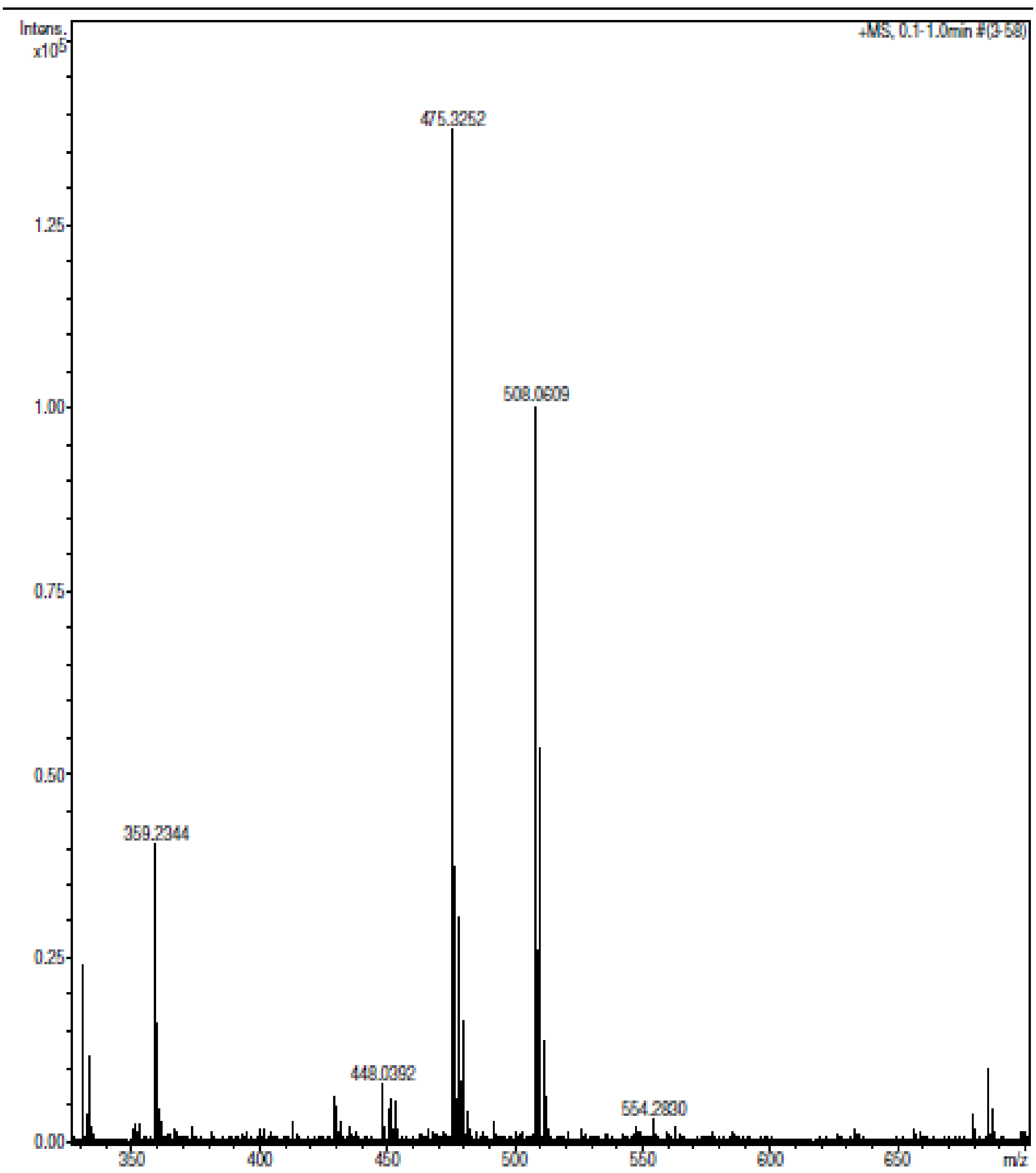

(c) ESI-Mass spectra of Curcumin-Cu(II) complex.

(d)

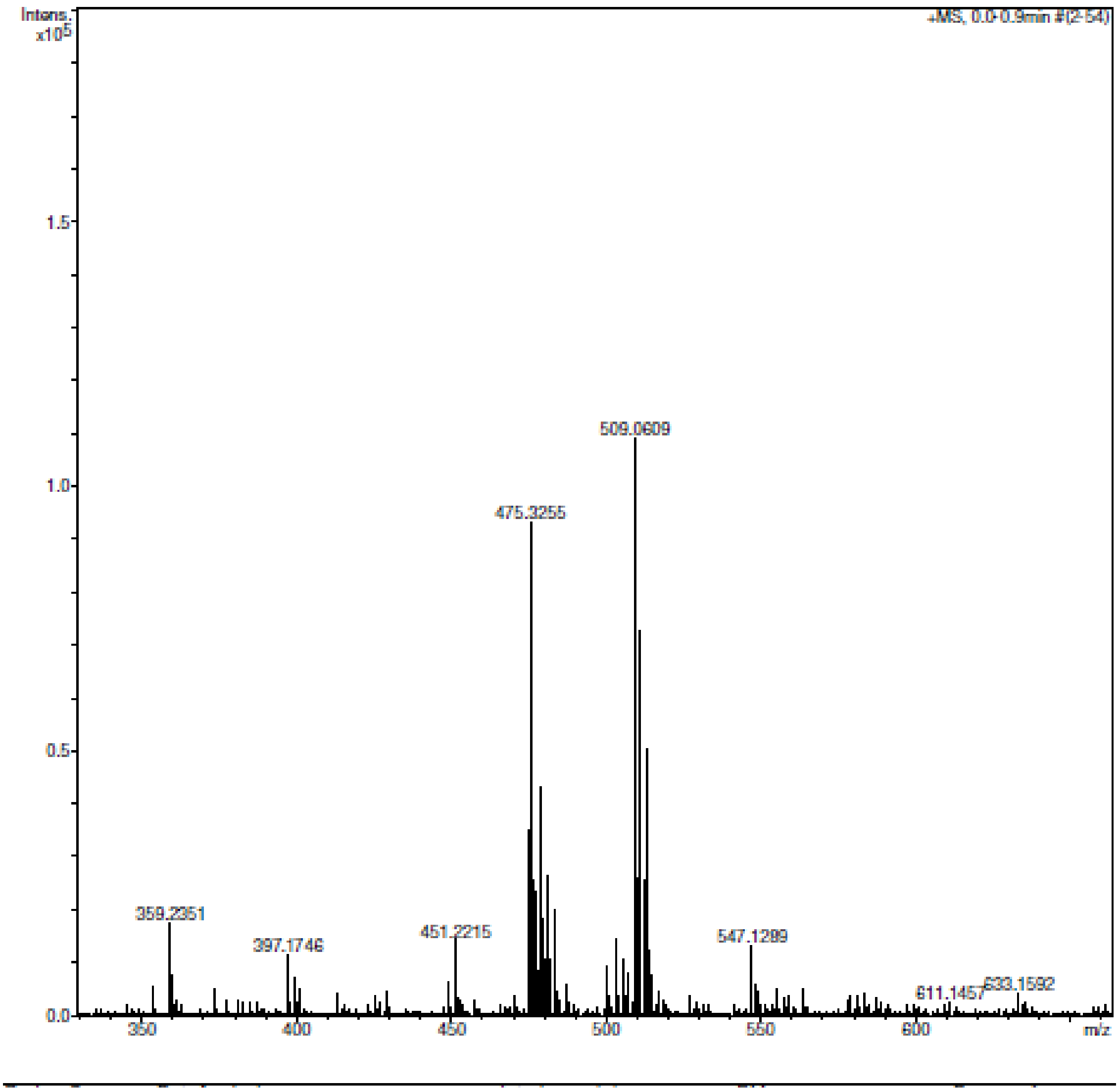

(d) ESI-Mass spectra of Curcumin-Zn(II) complex.
